# Infectious diseases during the European Union training mission Mali (EUTM MLI) – a four-year experience

**DOI:** 10.1186/s40779-018-0166-5

**Published:** 2018-05-31

**Authors:** Hagen Frickmann, Ralf Matthias Hagen, Florian Geiselbrechtinger, Nagpal Hoysal

**Affiliations:** 1 Department of Microbiology and Hospital Hygiene, Bundeswehr Hospital Hamburg, Bernhard Nocht Str. 74, 20359 Hamburg, Germany; 2 Institute for Medical Microbiology, Virology and Hygiene, University Medicine Rostock, Schillingallee 70, 18057 Rostock, Germany; 3 Department of Preventive Medicine, Bundeswehr Medical Academy, Neuherbergstraße 11, 80937 Munich, Germany; 4 NATO Center of Excellence for Military Medicine (MilMedCOE), Deployment Health Surveillance Capability (DHSC), Dachauer Str. 128, 80637 Munich, Germany; 5 Institute of Medical Information Processing, Biometry and Epidemiology, LMU Munich, Marchionini-Str. 15, 81377 Munich, Germany

**Keywords:** Tropical deployment, Infectious diseases, Tropical medicine, Gastrointestinal infections, Upper respiratory tract infections, Mali

## Abstract

**Background:**

The European Union Training Mission Mali (EUTM MLI) is a multinational military training deployment to the Western African tropical nation of Mali. Based on routinely collected disease and non-battle injury surveillance data, this study quantifies the true impact of infectious diseases for this tropical mission and potential seasonal variations in infectious disease threats.

**Methods:**

Categorized health events during the EUTM MLI mission and associated lost working days were reported using the EpiNATO-2 report. Infection-related health events were descriptively analyzed for a 4-year period from the 12th week in 2013 to the 13th week in 2017. Aggregated EpiNATO-2 data collected from all missions other than EUTM MLI were used as a comparator.

**Results:**

Among the infectious diseases reported by EUTM MLI, non-severe upper respiratory infections and gastrointestinal diseases dominated quantitatively, accounting for 1.65 and 1.42 consultations per 100 person-weeks, respectively. The number of recorded infectious disease-associated lost working days during the whole study interval was 723. Seasonal changes in disease frequency were detectable. More gastrointestinal infections were seen in the rainy season, and more respiratory infections occurred in the dry season; these were associated with peaks of more than 2.5 consultations per 100 person-weeks for both categories.

**Conclusions:**

Despite initial concerns focused on tropical infectious diseases during this mission in tropical Mali, upper respiratory tract and gastrointestinal infections predominate. The relatively low number of reported lost working days may indicate that these infections are at the milder end of the spectrum of infectious diseases despite a likely reporting bias.

**Electronic supplementary material:**

The online version of this article (10.1186/s40779-018-0166-5) contains supplementary material, which is available to authorized users.

## Background

In 2013, the European Training Mission in Mali (EUTM MLI) was initiated to support the military training of the Malian Armed Forces. EUTM MLI is a relatively small mission with less than 1000 European soldiers deployed to the topical environment of the south of the Republic of Mali. The aim of the mission is military on-site training of the armed forces of Mali. The military medical infrastructure comprises a role 1 unit at the mission headquarters in the capital Bamako and a role 2 unit at the major training site in Koulikoro.

General assumptions about the threat to the deployed force from tropical diseases and the general lack of reliable surveillance data in conflict areas led to the deployment of experts in tropical medicine and infectious diseases as part of the in-theatre medical support package at the beginning of the mission. Further, in collaboration with the Deployment Health Surveillance Capability (DHSC) of the NATO Centre of Excellence for Military Medicine, the health of the force deployed to EUTM MLI has been monitored continuously by using the EpiNATO-2 weekly surveillance report. This has been in place since the beginning of the mission.

EpiNATO-2 is the current and only interoperable illness and injury surveillance system within the Alliance. It has been in use since the twelfth calendar week of 2013, and EUTM MLI was one of the two early adopters of the system. The prospective EpiNATO-2 data collection is routinely analyzed by DHSC to provide timely information to the deployed commander about trends in illness and injury. Providers can report laboratory-confirmed disease in their weekly reports; however, the limited spectrum and availability of rapid near-patient diagnostics means that the focus of EpiNATO-2 is to monitor clinically diagnosed disease.

The authors are unaware of any contemporary published reports of prospective cohort data describing the epidemiology and burden of communicable infectious diseases in military personnel deployed to West Africa. Despite reconnaissance, the scarcity of available epidemiological data meant that a comprehensive causality estimate was not conducted prior to the mission. The health threats to travelers is well described in previous assessments [1–4], including some tailored for military audiences [5]. Tropical infectious diseases predominate, a finding reinforced in case reports of outbreaks, medical repatriations, and illnesses in returning travelers. In a French assessment of soldiers medically repatriated from Africa, *Plasmodium falciparum* malaria was by far the most frequent diagnosis [6]. Malaria was also identified as a major cause of severe infections during British deployments to Western African Sierra Leone [7–9]. The French army even reported one or two fatal courses of malaria per year on deployment [10].

EpiNATO-2 data collection provides a potential means of addressing this gap in knowledge. These data, together with occasional point prevalence assessments [11, 12], allow for a more realistic estimation of the actual burden of infectious disease threats. In this paper, we describe the patterns of infectious disease threats reported in EUTM MLI and relate this to a microbiological assessment of routinely collected food samples.

## Methods

### Data collection

The medical facilities in Bamako, Mali (role 1) and Koulikoro, Mali (role 2), reported consultations by military personnel deployed to the EUTM MLI mission weekly using the EpiNATO-2 illness and injury surveillance report. The surveillance method is described in detail within STANAG 2535 (Standardization Agreement), AMedP-4.1 (Allied Medical Publication) Deployment Health Surveillance, NATO 2017.

EpiNATO-2 monitors ambulatory care first attendances that can be categorized into one or more of 17 specified illness and injury events labelled Alpha through Quebec. These events were selected by a multinational expert panel for their relevance in terms of impact on operational effectiveness. A further non-specific event labelled Romeo is used to monitor unusual or exceptional events. All of the categorized events together with any other first and follow-up attendances are also summarized in a catch-all count of total consultations.

The clear majority of communicable infectious diseases are monitored using the 6 specified events described in Table 1 below. These are intended to monitor clinical diagnosis only. Where some form of laboratory confirmation is available, including rapid diagnostics, the case is reported in a notifiable infectious disease report. This provides a count of cases only and does not replace national notification procedures. Given that the primary source of surveillance data is from the outpatient role 1 medical treatment facilities, DHSC believes that almost all incident infectious disease is captured in the illness and injury surveillance report.

**Table 1:** Communicable infectious diseases monitored by EpiNATO-2

Event serial and name	Definition
Alpha – gastrointestinal illness (GI infections)	All diagnoses consistent with upper or lower digestive tract infection. Includes any type of diarrhea, gastroenteritis, “stomach flu”, “food poisoning”, nausea/vomiting, etc. Excludes non-infectious intestinal diagnoses such as hemorrhoids, ulcers, hernias, etc., and chronic conditions such as irritable bowel syndrome
Bravo – upper respiratory tract infection (URTI)	Acute upper respiratory infections of the ear, nose and throat. Includes “common cold”, tonsillitis, otitis, sinusitis, isolated acute laryngitis and tracheitis. Excludes acute laryngitis and tracheitis associated with an influenza-like illness (map to Item 4-Delta influenza-like illness and lower respiratory tract infection ILI). Excludes allergic or irritant conditions (if necessary, map to Item 18-Romeo unusual or exceptional event)
Delta - Influenza-like illness and lower respiratory tract infection (Flu symptoms and LRTI)	Illness characterized by fever (central temperature > 38.5 °C or > 101.3 °F) and either cough or sore throat, associated or not with laryngitis and tracheitis. Includes pneumonia and bronchitis
Echo – fever of unknown origin (Unexplained fever)	Central temperature > 38.5 °C or 101.3 °F, or history of chills and fever without a clear diagnosis. Such fever cannot be explained by other inflammatory or infectious processes such as respiratory infections, heat, or overexertion. Includes septicemia and viremia
Foxtrot – hemorrhagic illness	Acute systemic illness characterized by fever, chills, back pain or generalized myalgia and varying hemorrhagic manifestations such as bleeding gums, epistaxis, hematemesis, melena, metrorrhagia, strawberry tongue, disseminated intravascular coagulation, petechiae or bruising; consistent with viral hemorrhagic fever (VHF). Excludes biologically confirmed diagnosis of VHF – map to A.7 notifiable infectious diseases reporting
Hotel – acute neurological disorders	Acute infection or intoxication of the central nervous system (CNS). Includes meningitis, encephalitis, or encephalopathy and acute non-specific symptoms such as meningism and delirium. Excludes alcohol intoxication or any chronic, hereditary, or degenerative conditions of the CNS such as obstructive hydrocephalus, Parkinson’s, and Alzheimer’s.

It is likely that several cases of infectious disease will have been classified into other categories. For example, event Juliet is used to report dermatological disorders, and this includes fungal infections, cellulitis, and infestations. Furthermore, event Lima is used to report dental disorders that will include dental infections. A detailed study of cases reported in these events is needed to determine the proportion of reported cases that could be considered communicable.

It is also possible that some cases of infectious disease will be reported in event Romeo as an unusual or exceptional event. These cases should normally have an accompanying missive; however, very few accompanying notes have been received by DHSC. This and the aforementioned are limitations of the data collection, and the potential effect is under-estimation of the true communicable infectious disease burden. Notwithstanding, we believe that the 6 categories above capture the lion’s share of incident communicable infectious diseases.

Loss of productivity due to incident illness and injury in each of the specified events is a part of the EpiNATO-2 data collection. This is measured using lost working days (LWD), which is the sum of the days of complete exclusion from duties for all patients in a given specified event. Partial exclusions from duties are not monitored. In addition, there is no standardized way of prescribing exclusion from duties. Analysis published elsewhere [13] has shown heteroskedasticity or no obvious correlation between LWD and count of attendance where one might be expected a priori; therefore, the interpretation of these data is limited.

Data collected using the EpiNATO-2 format since ISO calendar week 12 of 2013 through calendar week 13 of 2017 is included in the analysis. Since week 14 of 2017, the EpiNATO-2 report from EUTM MLI has changed because of the publication of a new version of STANAG 2535. Broadly speaking, the changes simplify the language used in the original versions, and the intent is for future data to remain comparable with the past. The more significant changes were to remove event Romeo and add specified categories so that first attendances are now categorized exhaustively and to delete the reporting of LWD.

On deployment, the EpiNATO-2 data were certified by the role 1 physicians in charge. Physicians were provided with standard operating procedures by the theatre HQ (headquarters), which included reporting timelines, the definitions, and a reporting template. On a weekly basis, data were sent to the Medical Force Health Protection officer of the respective mission for a final consistency check. Afterwards, the data were forwarded to the DHSC for analysis and feedback of findings.

To assess potential differences with other deployments during the same assessment period, the data from EUTM MLI were compared with the aggregated EpiNATO-2 data collected from all other assessed operations being surveyed by DHSC. This includes operations in Kosovo, Afghanistan, Sierra Leone, Iraq, and the standing NATO maritime groups patrolling the Mediterranean.

### Data assessment

**Fig. 1:**
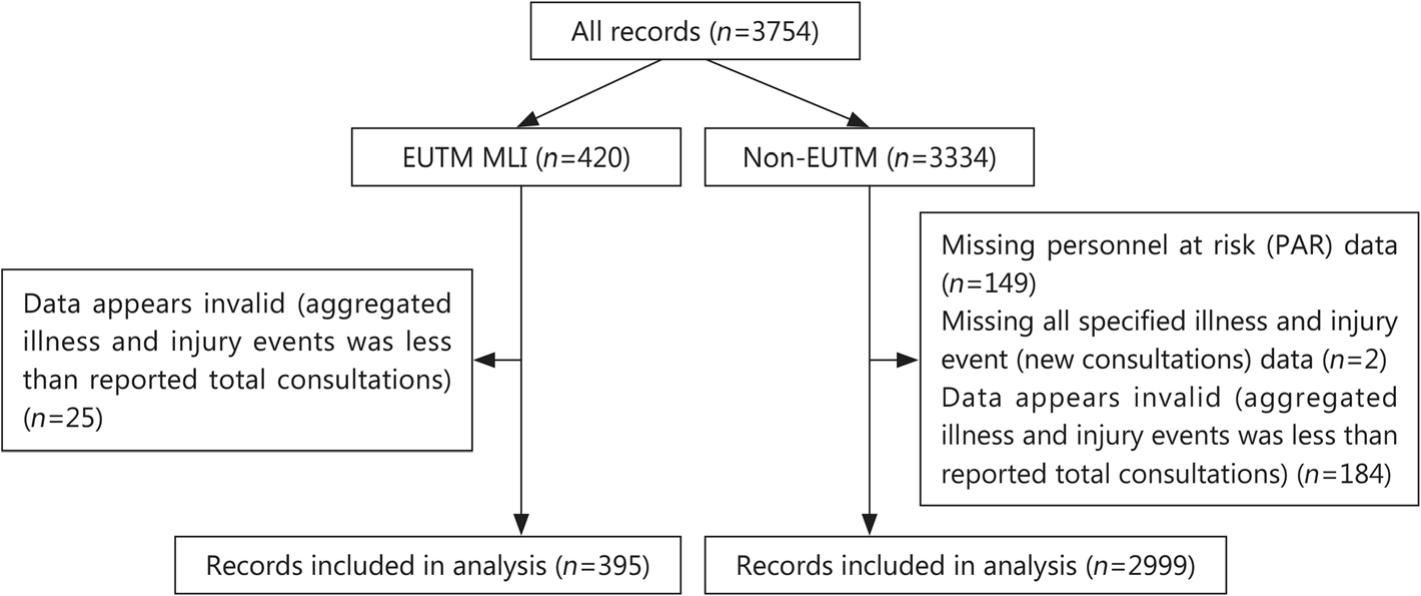
Results of data assessment and exclusion of records

The EpiNATO-2 reports included in the analysis were inspected to assess completeness and validity. Records were excluded if they were missing the number of personnel at risk, were missing all specified illness and injury events, or if the total number of consultations reported (which should include new and follow-ups) was less than the sum of the reported new cases of specified event categories. Figure 1 shows the results of the exclusion process. There were, however, no options to assess the completeness of entries in the databases.

### Microbiological real-life data from a hygiene screening in the canteen

As an etiological correlate of the observed gastrointestinal infections, the results from a microbiological assessment from a hotel canteen where EUTM MLI personnel regularly ate are presented as an example. The analysis was provided as an illustration to discuss practical implications of syndromic real-time surveillance. For this assessment, the analysis was restricted to qualitative assessment of bacterial growth due to the long transport and storage time of samples, which did not allow quantitative analysis.

Due to the expectation of inadequate storage and transport conditions on deployment, samples were taken with gel swabs (product number 80.1661.500, Sarstedt, Nümbrecht, Germany) to support the viability of bacteria. After swabbing, storage and transport of approximately three days at room temperature prior to delivery to a diagnostic laboratory in Germany was unavoidable due to logistic limitations. As quantitative assessment was not feasible under such poor pre-analytic conditions making considerable die-off of microorganisms likely [14], only qualitative assessment was deemed possible. Therefore, the swabs were incubated in non-selective thioglycolate broth (Oxoid, Wesel, Germany) at 36 °C for 24 h. Afterwards, culture and isolation were performed on non-selective Columbia sheep-blood agar (Oxoid) with subsequent differentiation and resistance testing using a VITEK II laboratory automate.

### Ethics

An ethical clearance was not considered necessary for this assessment, which is in line with national laws and regulations. The reason is that no personal-confidential data are presented, only case numbers provided by a super-national health authority and data from a microbiological assessment, which did not include any human samples.

## Results

### Data sets assessed during the study interval

The total denominator of the data collected during this interval was 1,148,565 person-weeks prior to applying the exclusion criteria. After excluding all records according to the criteria described above, 112,279 person-weeks for EUTM MLI and 957,341 for non-EUTM missions remained. For EUTM MLI there were 9805 new consultations reported using the EpiNATO-2 categories; 282 reports used category Romeo and thus were considered unusual or exceptional events. However, there was very little information about the underlying diseases of the Romeo incidents in the database. Accordingly, new cases and lost working days reported using this category were excluded from any further analysis.

### Descriptive assessment of the infectious disease-related categories

**Fig. 2:**
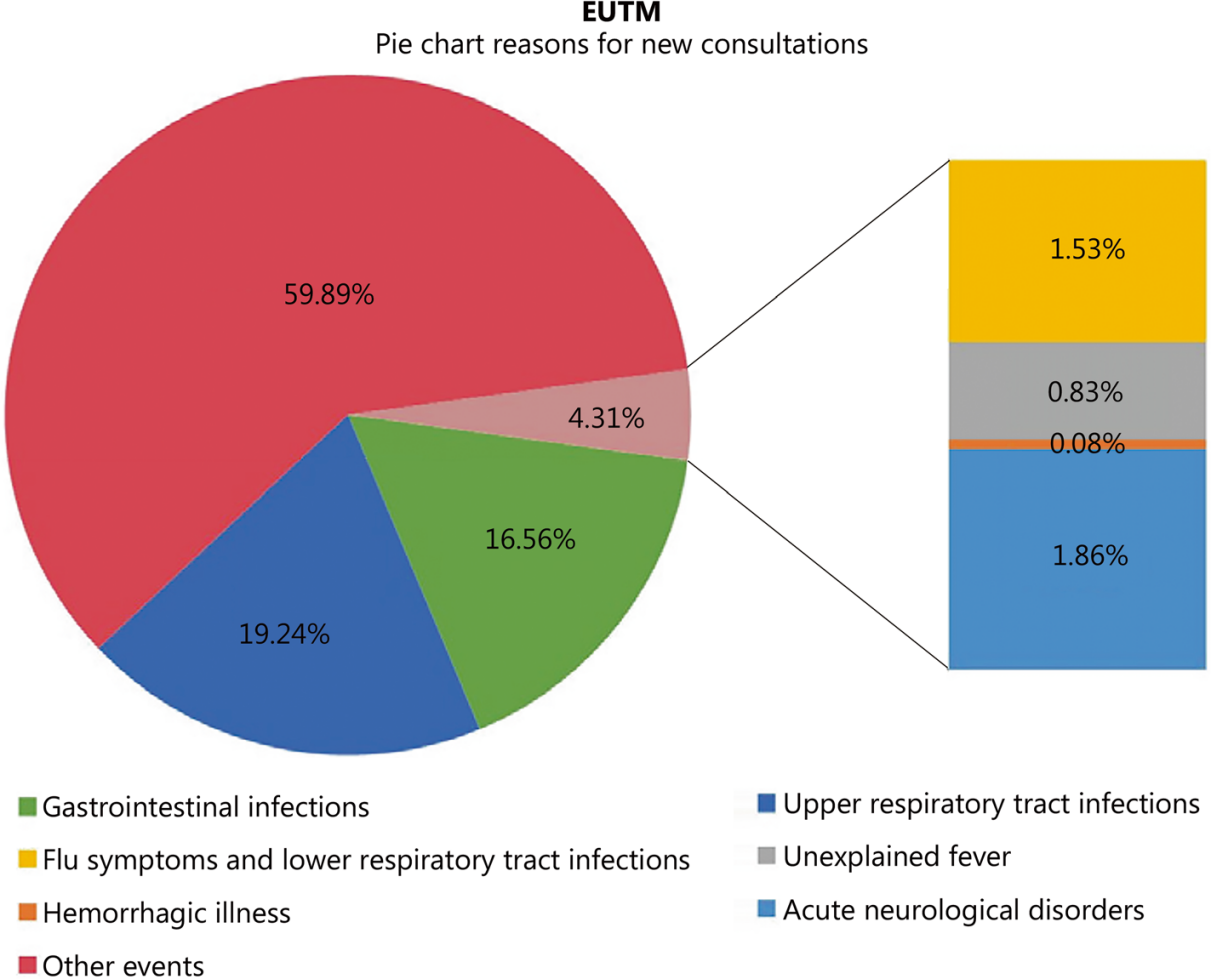
Most commonly reported reasons for new medical consultations during the study interval for EUTM MLI

The most commonly reported reasons for new consultations during the assessment period are shown in Fig. 2. The non-infectious diseases widely dominated, followed by URTI and GI infections. The number of cases over the study interval (and crude rate per person-week) of reported infectious disease-associated disease categories were as follows: GI infections: 1599 (1.42 per 100 person-weeks), URTI: 1858 (1.65 per 100 person-weeks), flu symptoms and LRTI: 148 (0.13 per 100 person-weeks), unexplained fever: 80 (0.25 per 100 person-weeks), hemorrhagic illness: 8 (0.01 per 100 person-weeks), and acute neurological disorders: 180 (0.16 per 100 person-weeks).

**Fig. 3:**
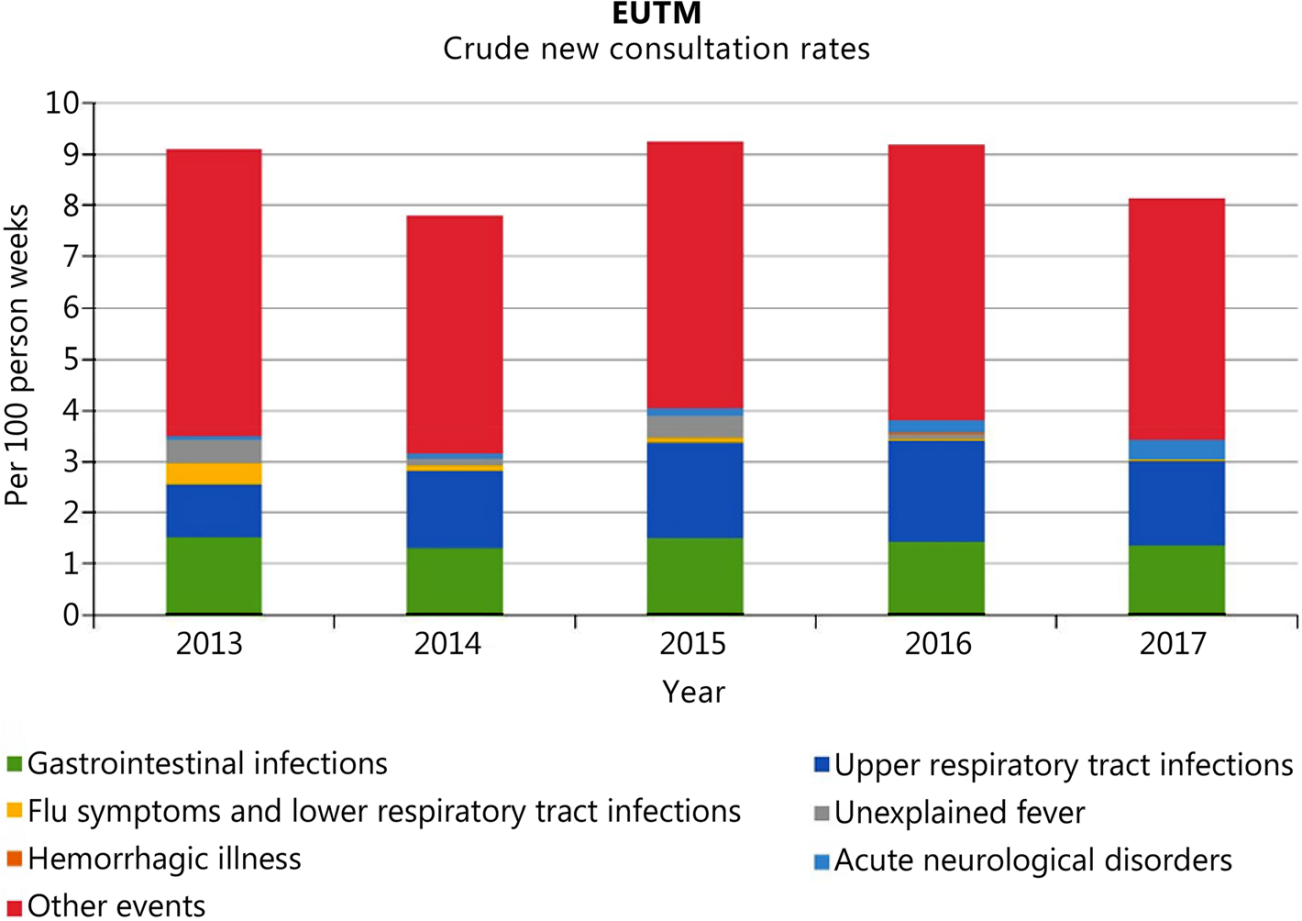
Variation in the crude rate of reported disease categories year by year for EUTM MLI

Figure 3 shows the variation in the crude rate of reported health events year by year. Over all assessed periods, the quantitative dominance of the URTI and GI infection events remained stable.

**Fig. 4:**
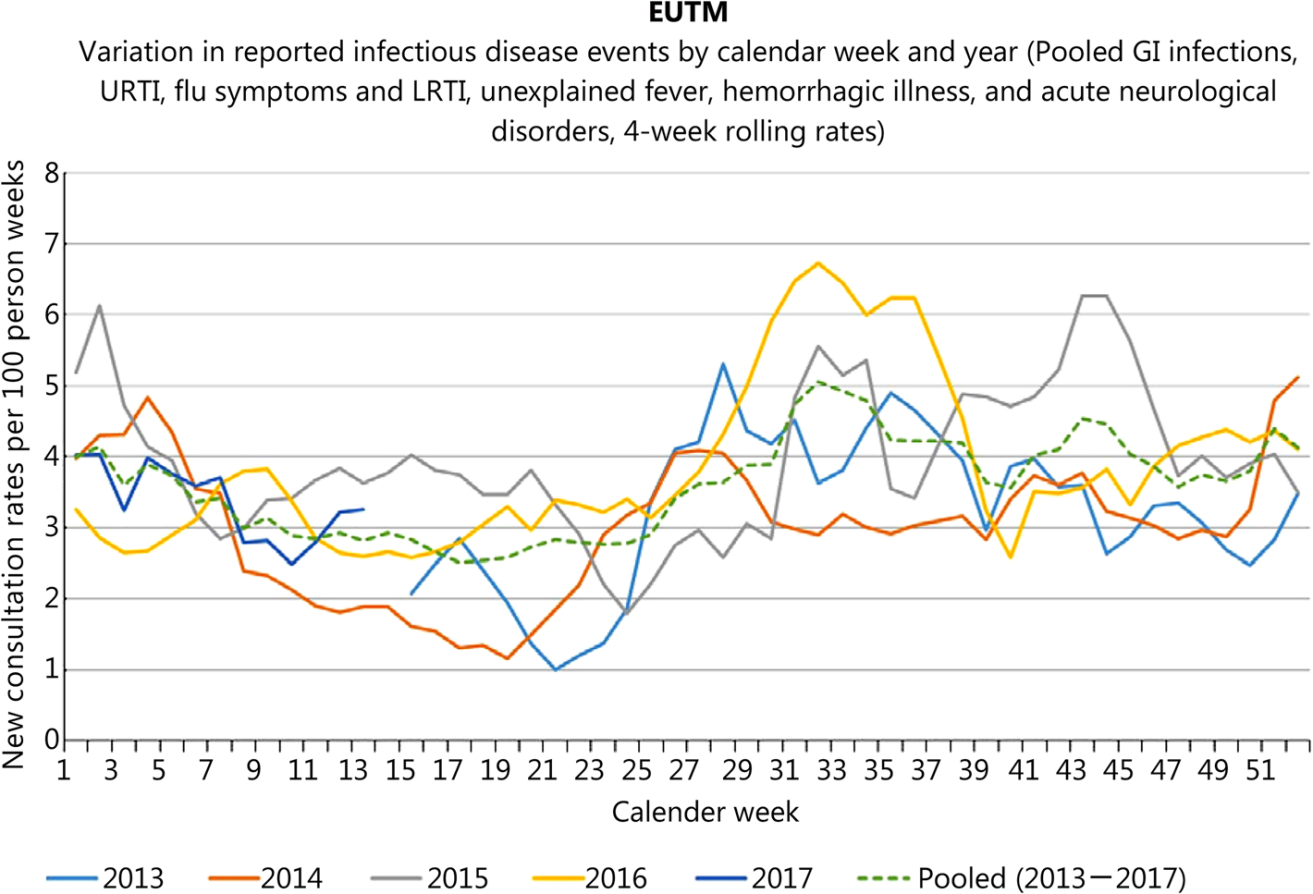
Variation in reported infectious disease events by category and year for EUTM MLI. Seasonal variation in new consultation rates by year (4-week rolling rates, reported infectious diseases only)

Figure 4 demonstrates the seasonal variation in the 4-week rolling average of the crude rate of reported infectious disease events, with the dashed line showing the pooled data over the whole study interval. The rate varied between slightly less than 2.5 and 5.0 consultations per 100 person-weeks with a slightly higher rate in the second half of the year with particularly pronounced occurrence at the end of the rainy season in summer. The values for CW 38 and 47 (2013), 9 (2014) and 40 (2015) were missing and therefore interpolated by calculating and rounding down the average of the values for the week before and the week after the missing values.

**Fig. 5:**
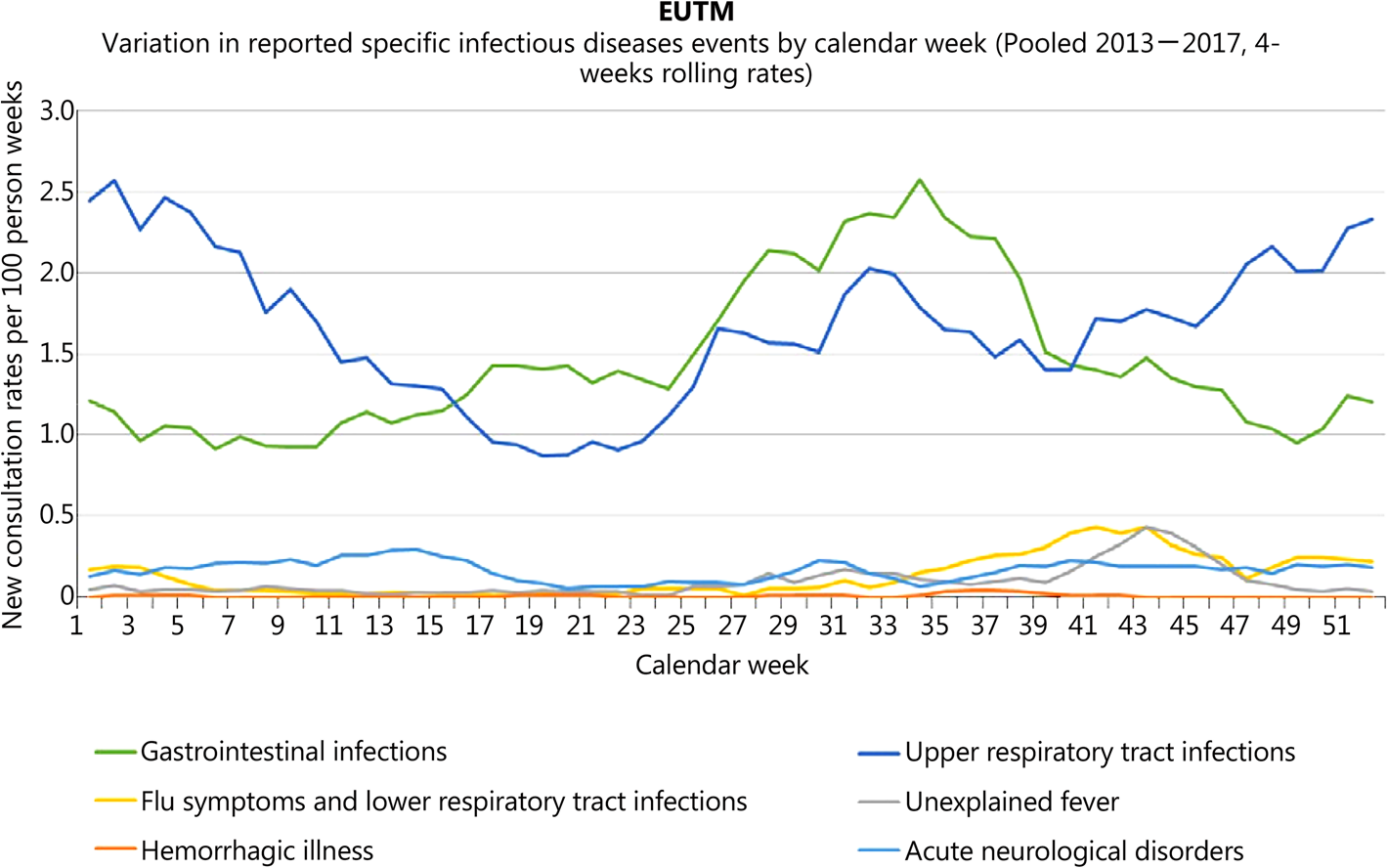
Variation in reported specific infectious diseases events by calendar week for EUTM MLI. Seasonal variation in new consultation rates by event (4-week rolling rates, reported infectious diseases only)

When making assessments on a category level, it becomes obvious that most of the variation is due to reporting of the URTI and GI infection events. The contribution of the categories to overall seasonal variation in infectious diseases reporting is shown in Fig. 5. While there is a marked increase in diarrhea in the rainy summer time, upper respiratory tract infections are more common in the dry season in winter.

Visual inspection suggests the rate of infectious diseases reported by EUTM MLI was consistently higher than for other missions (Additional file 1: Figures S1 and S2). Table 2 shows the results of the comparison of proportions using the “N-1”Chi-square test and confirms that there is significantly higher reporting of infectious diseases by EUTM MLI compared to the pooled rate for all non-EUTM missions. Compared with respiratory infections, gastrointestinal infections play only a minor role in non-EUTM theatres. The crude rate of reported infectious disease events on non-EUTM missions varied between slightly more than 1 and slightly more than 2 consultations per 100 person-weeks (Additional file 1: Figure S3), and there was no pronounced peak in the months of autumn. Focusing on disease categories, there is a slight increase in URTI events in the autumn and winter time and there are no marked variances for the other assessed categories (Additional file 1: Figure S4).

**Table 2:** Comparison of aggregated rates of EpiNATO-2 infectious disease events reported by EUTM MLI compared with non-EUTM missions

EpiNATO-2 event	Difference between EUTM and non-EUTM missions (%)	95% CI	Chi squared	df	*P* value
GI infections	0.9376	0.8677–1.0100	1519.222	1	< 0.0001
URTI	0.6661	0.5896–0.7449	425.692	1	< 0.0001
Flu symptoms and LRTI	0.1219	0.0968–0.1447	62.136	1	< 0.0001
Unexplained fever	0.0627	0.0297–0.0933	12.895	1	0.0003
Hemorrhagic illness	0.1800	0.0026–0.0088	0.593	1	0.4412
Acute neurological disorders	0.1293	0.1064–0.1547	377.100	1	0.0001
Other events	1.8967	1.7633–2.0323	1084.692	1	0.0001

*GI* Gastrointestinal, *URTI* Upper respiratory tract infections

**Fig. 6:**
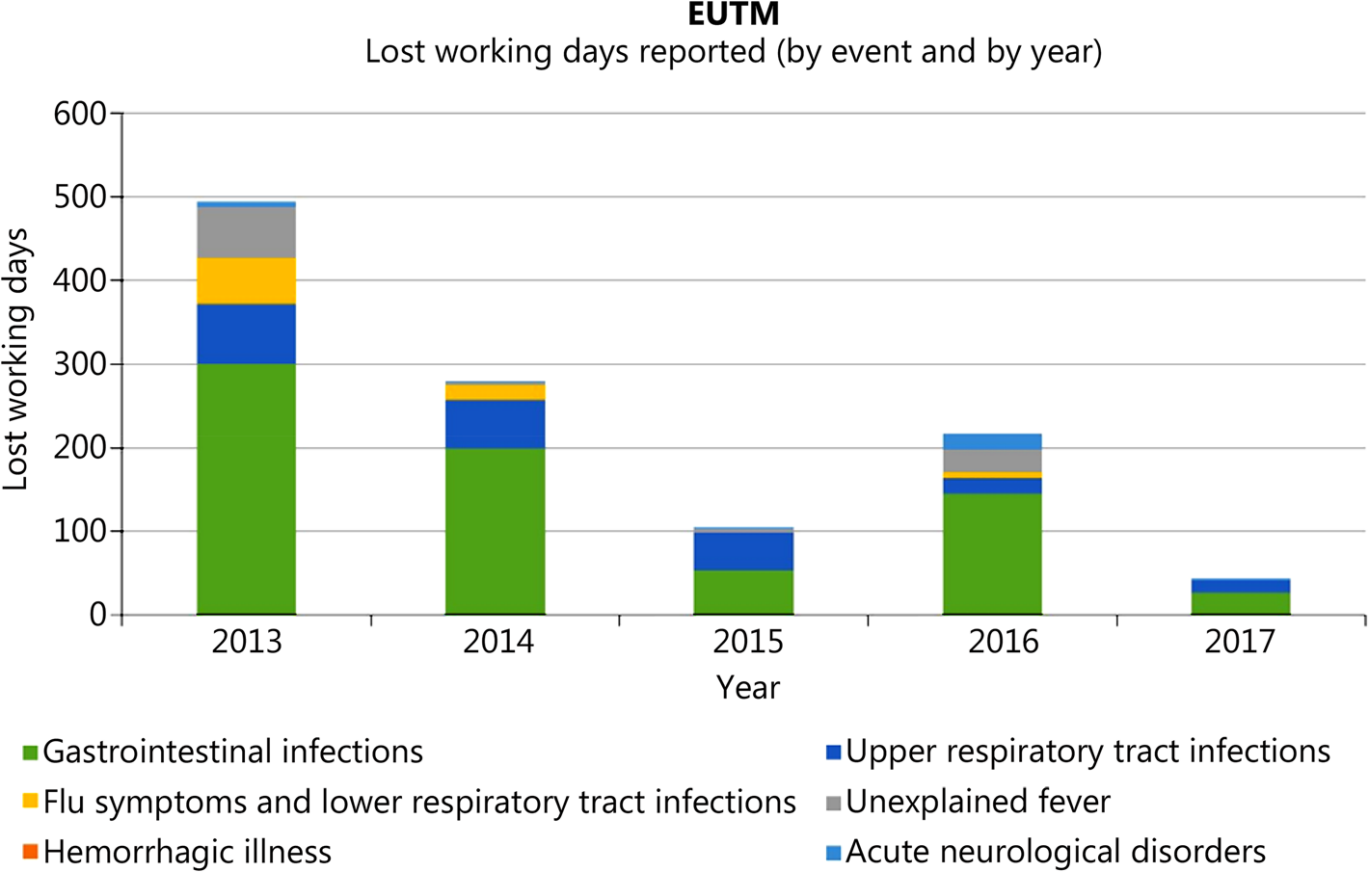
Lost working days during the study interval for EUTM MLI. Lost working days reported by event and by year

The number of lost working days by disease category is shown in Fig. 6. During the whole study interval, a total of 1837 lost working days has been reported for EUTM MLI. The number of reported lost working days declined from 4.4 per 100 person-weeks in 2013 to 0.93 per 100 person-weeks in 2017. There is no explanation for the decline in reported lost working days.

Of the reported lost working days, 700 were due to an infectious disease of the category GI infection (0.62 per 100 person-weeks). The second most common cause of lost working days was event category URTI, which contributed 205 lost working days (0.18 per 100 person-weeks). Given that the surveillance data source is ambulatory care consultations, the figures are likely to be an underestimate of the true impact in terms of loss of productivity. EpiNATO-2 does not monitor duration of hospital admissions or strategic aeromedical evacuations. Further investigation of this type of healthcare activity data may provide a better understanding of the true level of loss of productivity.

### Notifiable infectious diseases and events

Synchronized with the weekly EpiNATO-2 data collection, medical treatment facilities are required to submit a report of cases of infectious diseases where there is supportive or confirmatory laboratory evidence. This includes the results of rapid diagnostic tests deployed in the theatre. Reports of confirmed diseases in EUTM MLI during the study period are summarized in Additional file 1: Figure S5. The most common disease reported was malaria; however, the average number of cases during the entire study was only 0.02 per 100 person-weeks. The key limitation of these data is the lack of ascertainment of cases diagnosed following repatriation or after assessment of samples by national reference laboratories.

### Microbiological real-life data from a hygiene screening in the canteen

As the GI infection category was the main reason for lost working days due to infectious diseases, a microbiological assessment of the restaurant of the hotel, where the EUTM MLI headquarter was deployed, was conducted in the 4th quarter of the year 2015. The results are shown in Table 3. Fecal contamination could be non-quantitatively confirmed for both the provided food and for equipment and the infrastructure of the kitchen. Potentially disease-related microorganisms such as *Bacillus cereus* and extended-spectrum-beta-lactamase-(ESBL)-expressing *Enterobacteriaceae* were observed.

**Table 3:** Results for the microbiological screening in the hotel canteen by sample material and detected microorganisms

Analyzed material	*Bacillus cereus*	*Escherichia coli*	Enterococci	Further potentially harmful species
Cut papaya fruit from the breakfast buffet	Negative	Negative	Positive	*Pseudomonas aeruginosa*
Cut watermelon from the breakfast buffet	Negative	Negative	Positive	*Enterobacter cloacae* ssp. *cloacae*
Tomatoes in the hotel kitchen	Positive	Negative	Positive	ESBL-positive *Klebsiella oxytoca*, *Acinetobacter* spp.
Apples in the hotel kitchen	Positive	Negative	Negative	*Enterobacter cloacae* ssp. *cloacae*
Cut cabbage in the hotel kitchen	Negative	Negative	Negative	*Pseudomonas aeruginosa*, *Klebsiella pneumoniae*
Handle of the big refrigerator in the hotel kitchen	Negative	Negative	Positive	*Aeromonas hydrophila*/*caviae*
Cutting machine of the hotel kitchen	Negative	Negative	Positive	*Delftia acidovorans*, *Acinetobacter pittii*
Cutting plate of the hotel kitchen	Positive	Negative	Positive	*Enterobacter cloacae* ssp. *cloacae*, *Klebsiella pneumoniae*
Flour from a working place in the bakery	Negative	Negative	Negative	*Klebsiella oxytoca, Enterobacter cloacae* ssp. *cloacae*
Red chili sauce from the lunch buffet	Negative	Negative	Negative	*Enterobacter cloacae* ssp. *cloacae*, *Acinetobacter ursingii*
White rice from the lunch buffet	Negative	Negative	Negative	*Pantoea agglomerans*
Cut carrots from the lunch buffet	Negative	Negative	Positive	*Acinetobacter pittii*, *Aeromonas hydrophila/caviae*, *Kluyvera cryocrescens*
Cold chicken salad from lunch	Negative	Positive	Positive	*Aeromonas hydrophila/caviae*, *Enterobacter ludwigii*
Cake as a dessert from lunch	Negative	Negative	Negative	*Klebsiella pneumoniae*, *Enterobacter cloacae* ssp. *cloacae*, *Klebsiella oxytoca*
Paprika salad from the dinner buffet	Negative	Positive	Positive	*Pseudomonas aeruginosa,* *Proteus* spp.
Mayonnaise salad from the dinner buffet	Negative	Negative	Positive	*Pseudomonas aeruginosa,* *Proteus* spp.
Olives from the dinner buffet	Negative	Negative	Positive	*Bacillus cereus* var. *mycoides,* *Proteus* spp.

## Discussion

This assessment was performed to quantify the infectious disease burden during the EUTM MLI mission to allow a rational risk assessment for this tropical deployment. Tropical infectious diseases were considered a major health threat to the deployed personnel. Microbiological onsite surveillance data from EUTM MLI are scarce. In response to repeated complaints on the frequent occurrence of acute diarrhea episodes during this mission, a diagnostic surveillance was performed from December 2013 to August 2014 on this issue. Diarrheagenic *Escherichia coli* and noroviruses were identified as the most frequent causative agents, while invasive bacteria and protozoa were less frequent [11], and the diseases usually responded well to short-term antibiotic therapies with rifaximin, ciprofloxacin or azithromycine. Of note, considerable beta-lactame resistance in local *Escherichia coli* strains was observed as a side phenomenon [12].

To allow a broader assessment, clinical diagnosis surveillance data is presented in this study. Among the registered infectious diseases, there was a marked quantitative dominance of upper respiratory tract infections and gastrointestinal infections. The reporting of infectious diseases confirmed by laboratory results was very low and, of those reported, malaria was the most common.

An obvious seasonal variance, potentially associated with the shift of the dry season to the rainy season and vice versa, was only detectable for upper respiratory tract infections and gastrointestinal infections as well. One might speculate that the conditions during the rainy seasons might have facilitated both bacterial growth on warm moist surfaces, smear-associated pathogen transmission and spoiling of food, resulting in more diarrhea during this period, although underlying mechanisms are still poorly understood [15]. In contrast, the dry season in Mali is associated with winter season in Europe, when upper respiratory tract infections are more frequent [16]. The increased influx of disease aligns with changes in the mucous membranes during the dry season in Mali, as dry mucous membranes become further dehydrated by air-conditioning systems in the dry, hot weather. If there is poor maintenance for air-conditioning systems, they might further contribute to the spread of upper-respiratory disease by whirling up pathogens from contaminated filters; in addition, the associated cooling of the nasal airway may be a facilitating factor, as previously discussed [17].

Interestingly, the number of lost working days due to infectious diseases was disproportionately lower than the actual number of cases. This might indicate a strong dominance of infections with low severity. Alternatively, the fact that the number of lost working days is considerably lower than the total number of health events reported may suggest underreporting. The marked decrease in the number of lost working days over the years, for which no specific reason was detectable, might support this hypothesis. It is also likely that lost working days were incompletely ascertained as the surveillance system does not monitor hospital admissions or strategic evacuation.

The low number of lost working days is also in contradiction with previous experience from other missions. The British, for example, reported an average of 2.8 lost working days per person due to gastrointestinal infections during operation HERRICK in Afghanistan [18]. Such comparisons make underreporting and incomplete ascertainment likely. However, to the authors’ experience from EUTM MLI, GI episodes were usually mild and did not necessarily result in exclusion from duty. To reduce nosocomial spread, strict precautions regarding basic and toilet hygiene were enforced that allowed the patients to remain on duty. Other than in battle operations, the physical efforts during the training mission in Mali were usually negligible, so operability of the forces could be maintained despite low-impact infections. Therefore, low numbers of lost working days due to mild infections is not basically implausible, although incomplete reporting is likely to contribute to the low numbers of recorded lost working days.

In comparison with other deployments in the assessment period, the proportion of gastrointestinal infections is markedly increased for the EUTM MLI mission. The total proportions of infectious diseases are increased as well and, more than this, no seasonal changes were observed for the averaged non-EUTM missions.

The strong dominance of gastrointestinal infections among the lost working days due to infectious causes correlates well with fecal contamination as observed in the food provided by the restaurant of the hotel where the headquarter of EUTM MLI was deployed. Long transport and storage time of samples did not allow a quantitative assessment of pathogens, an undeniable limitation. In fact, the analysis was not performed to deduce justiciable consequences from the results but, instead, to get a crude estimation on the physical presence or absence of potential pathogens and fecal contamination in a quantity being sufficient to allow for cultural detection even despite inadequately long storage and transport time. Due to these modest demands, basically inacceptable deviations, both from national screening standards and the standards by the Codex Alimentarius of the World Health Organization, were accepted due to reduced logistic options that were mission-associated. Although the assessment led to some infrastructure changes in the kitchen of the restaurant, the effects of these procedures remained questionable, and a control assessment could not have been performed for logistic reasons. A change from food consumption in the hotel to definitely safe food sources such as military field rations was avoided for operational reasons, although the safety of military field rations was confirmed by previous diarrhea surveillance in Mali, where no diseased individual had consumed only such rations [11]. The operational reasons included the need for joint food consumption together with the local allies in their local infrastructure in Mali during meetings for most of the soldiers from the headquarters. Accordingly, a step-wise adaptation to local hygiene conditions and food-associated contamination seemed advisable to mitigate associated health-related consequences in the intermediate term [19]. Further, consumption of local food outside the camp could not have been suppressed due to relatively free movements of the soldiers as part of the mission policy. Accordingly, a baseline diarrhea rate that remained within ranges that seemed acceptable to the military mission command and did not lead to unacceptably high numbers of lost working days was tolerated [19].

Among the overwhelming proportion of non-severe infectious diseases, the identification of severe cases potentially requiring immediate action may be a challenge for the field doctor in charge and requires constant awareness during tropical deployment. In line with this, there was no hint for a detectable increase of disease categories suggesting severity, for example, severe respiratory tract infections, fever, hemorrhage and neurological disorders, in the rainy season. Therefore, the attentiveness for such events requires the same level during the whole year. Fortunately, there have been no hints for a high frequency so far. As stated above, unusual or exceptional events of the category Romeo were, however, not included in the assessment due to poor available data.

Despite the obviously low severity of the frequently detected gastrointestinal and respiratory infections, their calculated therapy remains challenging due to a broad differential diagnostic spectrum. This could be shown by surveillance on diarrhea-associated pathogens in the same mission [11]. Broad spectrum antibiotic drugs such as macrolides and fluoroquinolones have shown good effects despite this differential diagnostic uncertainty, as previously demonstrated [20–22] and documented in guidelines [23, 24]. However, broad spectrum antibiotic drugs are likely to support the selection of multidrug resistant bacteria [25]. Information on the local resistance situation was scarce, although point prevalence assessments suggested an abundance of ESBL-positive *Enterobacteriaceae* [12]. Fortunately, respective transient colonization of the soldiers’ gut seems to be of short duration, and the enteric carrier rate of German soldiers with ESBL-positive strains at 8–12 weeks after deployment is low [26].

Solutions for the field doctor should include low-threshold availability of options for infectious disease diagnostics in theatre [27] and sustained enforcement of basic hygiene procedures for military field camps in the tropics.

Limitations of the study comprise potentially varying qualifications and motivations of the field doctors who had to enter the EpiNATO-2 data. Although this source of bias is difficult to quantify, it might explain phenomena such as the low documented number of lost working days. Thus, database entries were vulnerable to random errors. More than this, syndromic surveillance is poorly suited to identify minor changes of rare but potentially severe diseases. Other sources of information, such as reports from aeromedical repatriation, are more likely to identify the frequency of rare but severe diseases such as those demonstrated by other groups [28, 29]. However, aeromedical repatriation is in the national responsibility.

Accordingly, respective data were not available for this super-national assessment, and a respective analysis would have been beyond the scope of this work. Finally, data from syndromic surveillance can only represent ill soldiers who present to medical care. For low impact health conditions, individuals may treat themselves instead of seeking medical care, resulting in underreporting, as suggested by others based on troop surveys [18, 30, 31].

## Conclusions

Although infectious diseases are frequent during the EUTM MLI mission in tropical Mali, the infections mainly comprise non-severe gastrointestinal and respiratory diseases. The associated number of lost working days is low; however, this is likely to be due to incomplete ascertainment and under-reporting. The quantitative proportions of flu symptoms and lower respiratory tract infections, unexplained fever, hemorrhagic illness, and acute neurological disorders were very low compared to gastrointestinal infections and upper respiratory tract infections. This suggests that an in-theatre specialist in tropical infectious diseases may not be required and a generalist or community physician is adequate; however, the field doctor would still need support in the form of reach back to this type of subject matter expertise. Near-patient diagnostic tools may facilitate the differential diagnosis for the field doctor in charge. Notwithstanding, the reinforcement of basic field hygiene and sanitation measures is still relevant now, as it always has been, in military field camps on tropical deployments.

## Additional file

Additional file 1: **Figure S1.** Most commonly reported reasons for new medical consultations during the study interval for non-EUTM missions. **Figure S2.** Variation in the crude rate of reported disease categories year by year for non-EUTM missions. **Figure S3.** Variation in reported infectious disease events by category and year for non-EUTM missions. **Figure S4.** Variation in reported specific infectious diseases events by calendar week for non-EUTM missions. **Figure S5.** Reports of confirmed diseases in EUTM MLI during the study period. (DOC 2979 kb)

## Abbreviations

AMedP: Allied medical publication
CNS: Central nervous system
DHSC: Deployment Health Surveillance Capability
ESBL: Extended-spectrum beta-lactamase
EUTM MLI: European Union Training Mission Mali
GI: Gastrointestinal
HQ: Headquarters
ILI: Influenza-like illness
LRTI: Lower respiratory tract infections
LWD: Lost working day
MilMedCOE: Center of excellence for military medicine
NATO: North Atlantic Treaty Organization
PAR: Personnel at risk
URTI: Upper respiratory tract infections
VHF: Viral hemorrhagic fever
