# Supplementary material for: Infectious diseases during the European Union training mission Mali (EUTM MLI) – a four-year experience
Source: Mil Med Res. 2018 May 31;5:19. doi: 10.1186/s40779-018-0166-5 (PMC5977544; doi:10.1186/s40779-018-0166-5)

Figure S1. Most commonly reported reasons for new medical consultations during the study interval for non-EUTM missions.


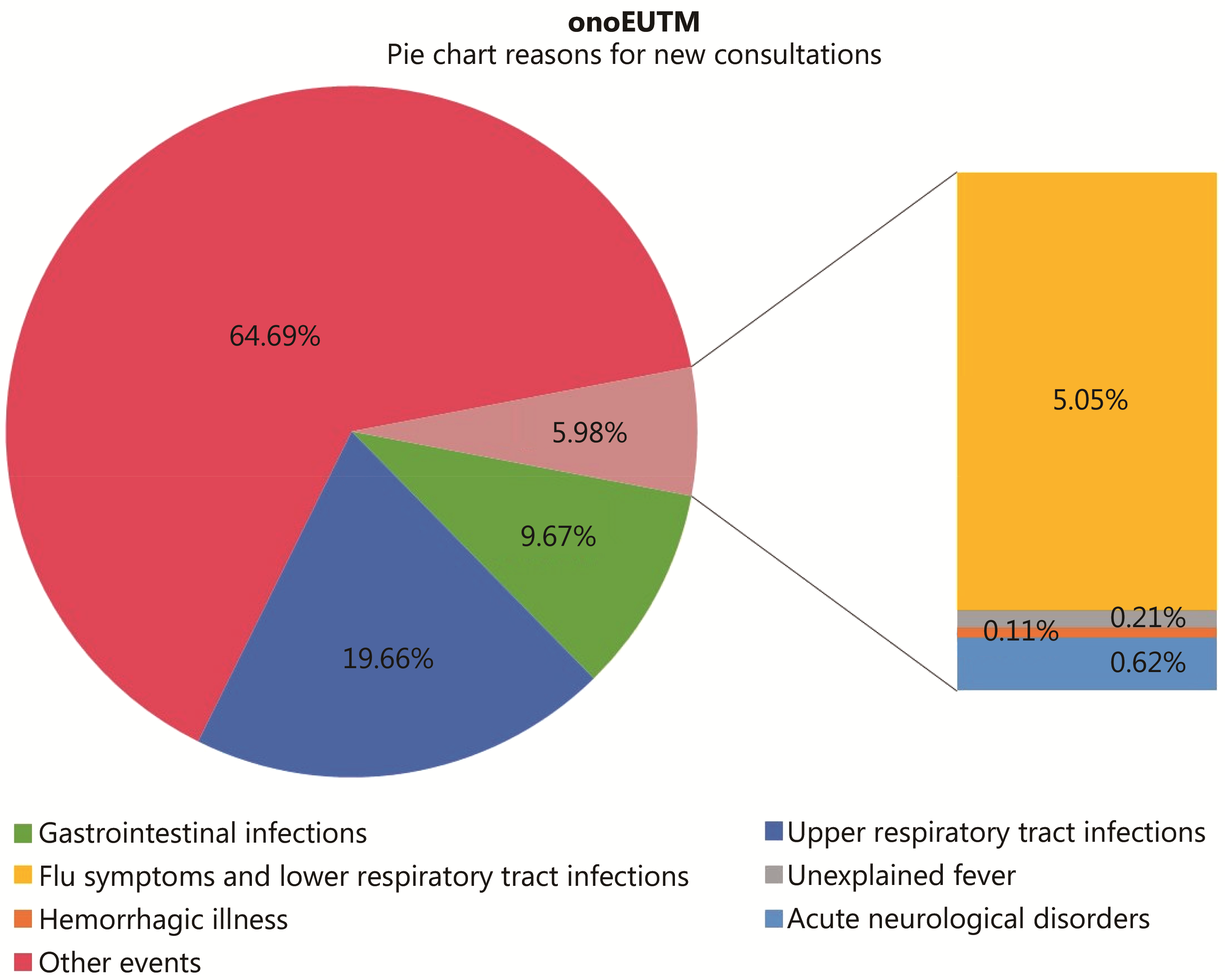


Figure S2. Variation in the crude rate of reported disease categories year by year for non-EUTM missions.


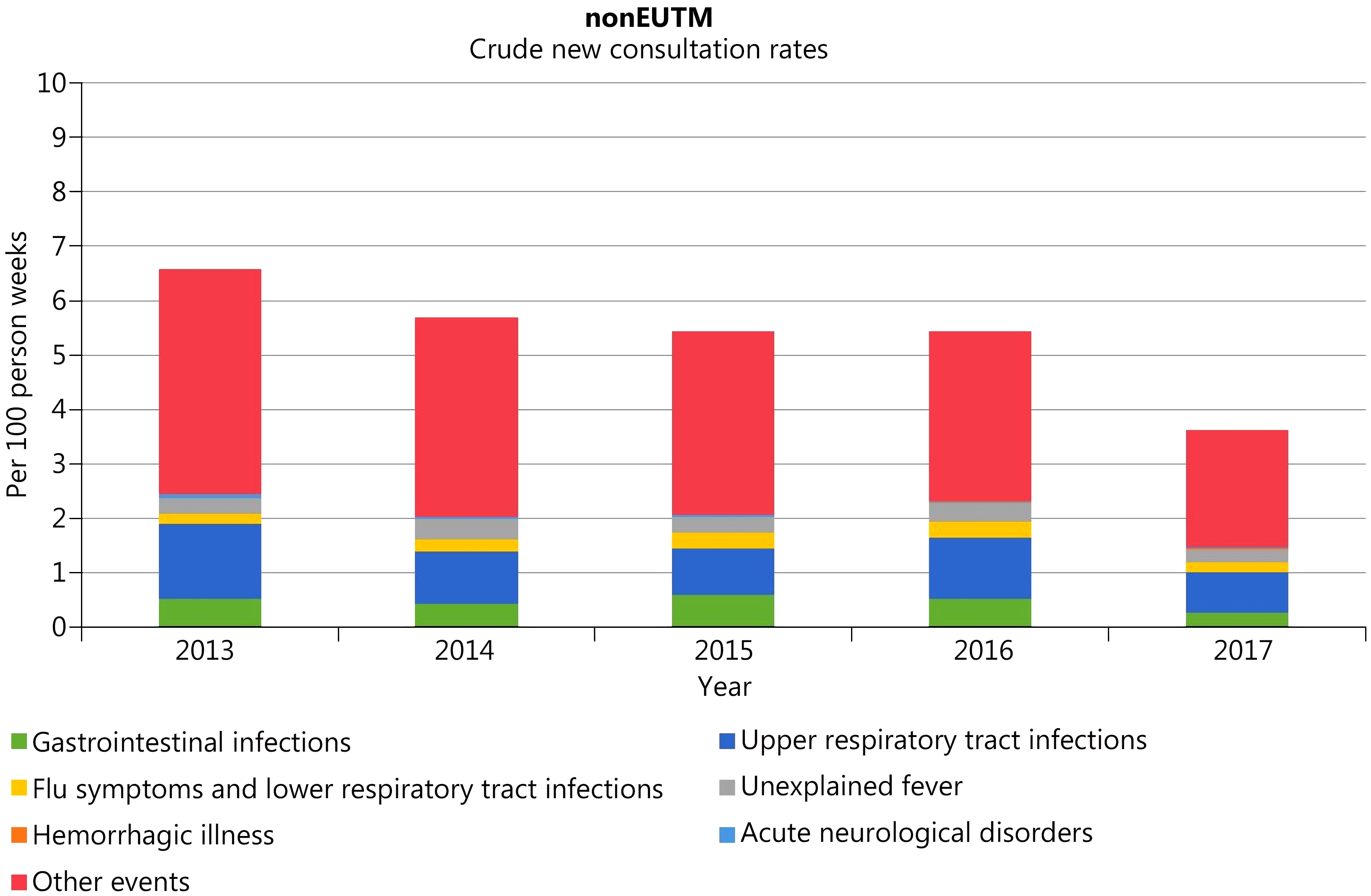


Figure S3. Variation in reported infectious disease events by category and year non-EUTM missions.


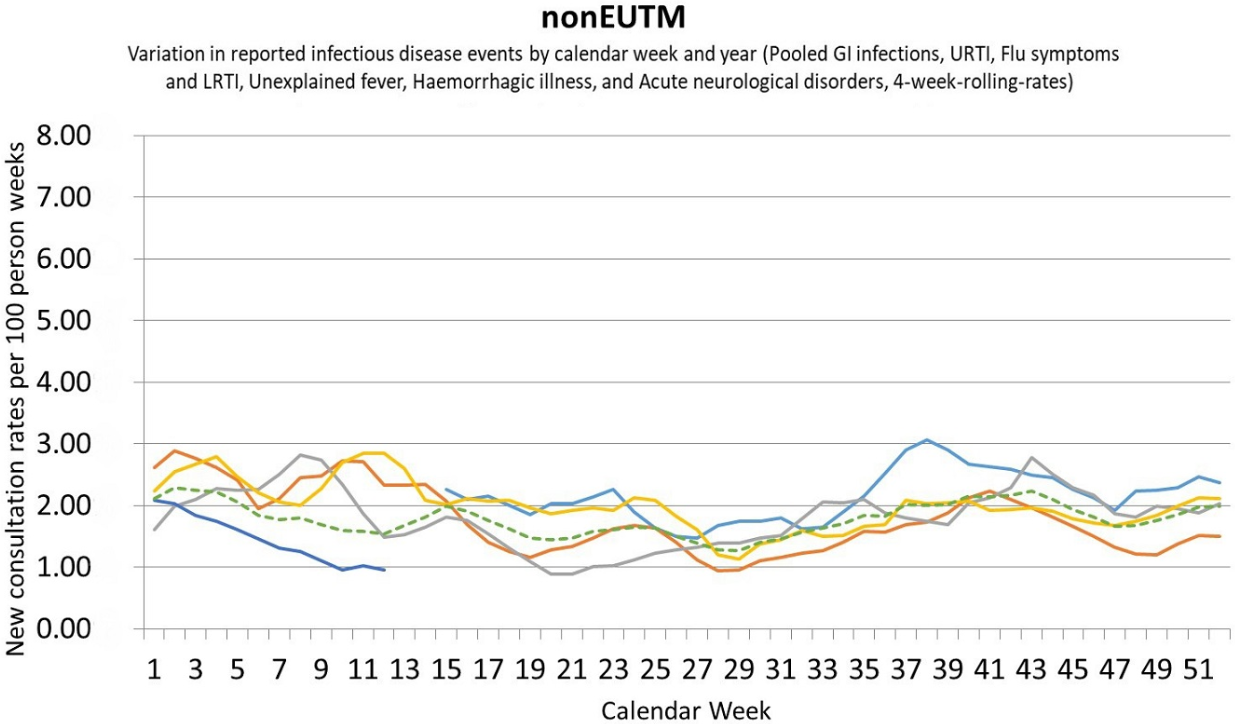


Figure S4. Variation in reported specific infectious diseases events by calendar week for non-EUTM missions.


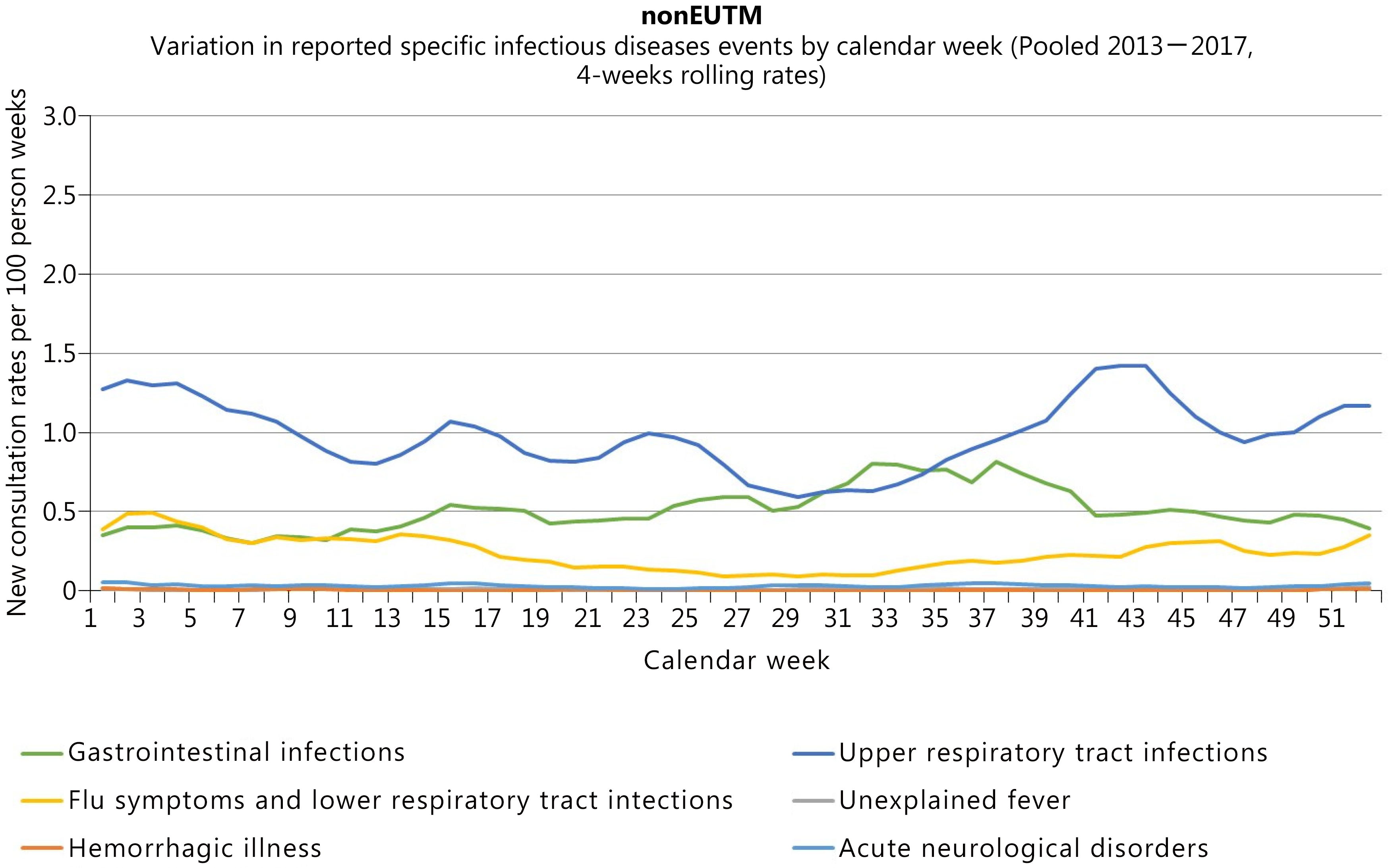


Figure S5. Reports of confirmed disease in EUTM MLI during the study period.


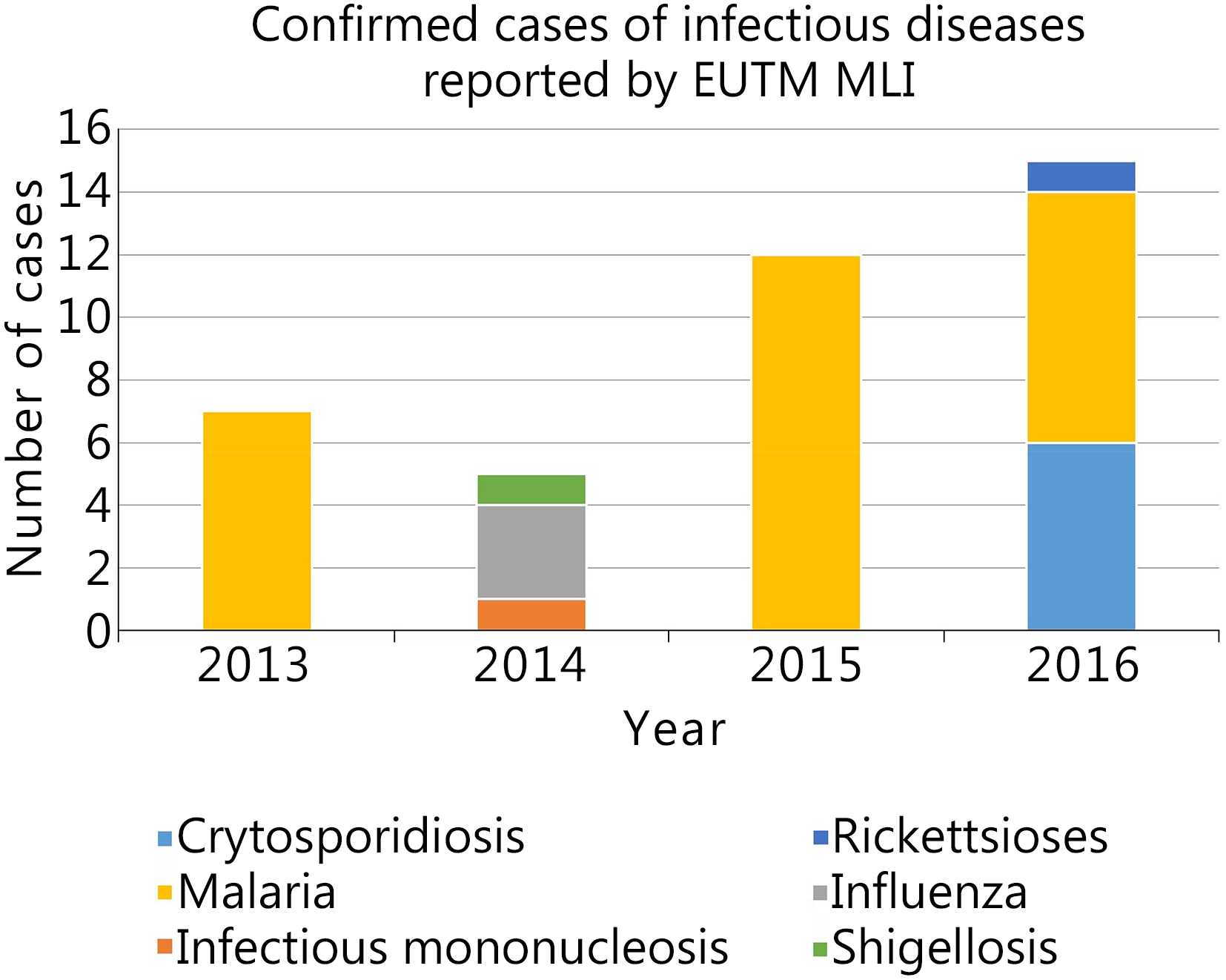

Supplement: Supplementary file 1 — Figure S1. Most commonly reported reasons for new medical consultations during the study interval for non-EUTM missions. Figure S2. Variation in the crude rate of reported disease categories year by year for non-EUTM missions. Figure S3. Variation in reported infectious disease events by category and year for non-EUTM missions. Figure S4. Variation in reported specific infectious diseases events by calendar week for non-EUTM missions. Figure S5. Reports of confirmed diseases in EUTM MLI during the study period. (DOC 2979 kb) [file 40779_2018_166_MOESM1_ESM.doc]
